# Regulatory factors governing adenosine-to-inosine (A-to-I) RNA editing

**DOI:** 10.1042/BSR20140190

**Published:** 2015-03-31

**Authors:** HuiQi Hong, Jaymie Siqi Lin, Leilei Chen

**Affiliations:** *Cancer Science Institute of Singapore, National University of Singapore, Singapore 117599, Singapore; †Department of Anatomy, Yong Loo Lin School of Medicine, National University of Singapore, Singapore 117597, Singapore

**Keywords:** adenosine deaminases acting on RNA (ADARs), adenosine-to-inosine (A-to-I) RNA editing, auto-regulation, post-translational modification, self-editing, transregulation, 5HT_2C_R, serotonin-2C receptor, ADAR, adenosine deaminase acting on RNA, A-to-I, adenosine-to-inosine, CREB1, cAMP responsive element-binding protein 1, dsRBD, dsRNA-binding domain, GluR-B, glutamate-gated ion channel receptor, NES, nuclear export signal, NLS, nuclear localization signal, Pin1, Phosphorylation-dependent prolyl-isomerase, SUMO, small ubiquitin-like modifier, VA, virus-associated

## Abstract

Adenosine-to-inosine (A-to-I) RNA editing, the most prevalent mode of transcript modification in higher eukaryotes, is catalysed by the adenosine deaminases acting on RNA (ADARs). A-to-I editing imposes an additional layer of gene regulation as it dictates various aspects of RNA metabolism, including RNA folding, processing, localization and degradation. Furthermore, editing events in exonic regions contribute to proteome diversity as translational machinery decodes inosine as guanosine. Although it has been demonstrated that dysregulated A-to-I editing contributes to various diseases, the precise regulatory mechanisms governing this critical cellular process have yet to be fully elucidated. However, integration of previous studies revealed that regulation of A-to-I editing is multifaceted, weaving an intricate network of auto- and transregulations, including the involvement of virus-originated factors like adenovirus-associated RNA. Taken together, it is apparent that tipping of any regulatory components will have profound effects on A-to-I editing, which in turn contributes to both normal and aberrant physiological conditions. A complete understanding of this intricate regulatory network may ultimately be translated into new therapeutic strategies against diseases driven by perturbed RNA editing events. Herein, we review the current state of knowledge on the regulatory mechanisms governing A-to-I editing and propose the role of other co-factors that may be involved in this complex regulatory process.

## Abstract 2

This review discusses the current state of knowledge on the mechanisms adopted in regulation of Adenosine-to-Inosine RNA editing.

## INTRODUCTION

**Figure 1:**
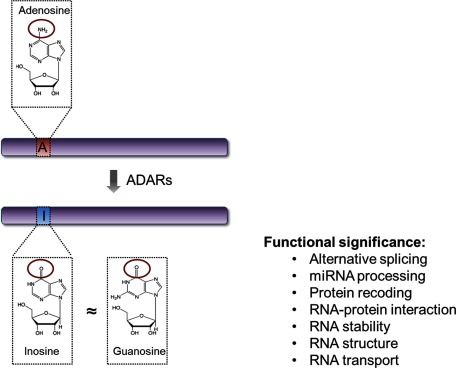
ADARs catalyse hydrolytic deamination of adenosines to inosines General cellular machineries decode inosine as guanosine, as inosine structurally resembles guanosine. This essentially introduces nucleotide change and contributes to transcript diversity. A-to-I editing is a significant post-transcriptional modification as it affects numerous cellular processes, including alternative splicing, miRNA processing, protein recoding, RNA–protein interaction, RNA stability, structure and transport.

Conversion of adenosine into inosine, otherwise known as A-to-I (adenosine-to-inosine) RNA editing, is catalysed by members of the adenosine deaminase acting on RNA (ADAR) family which act specifically on dsRNAs. A-to-I editing is a pivotal cellular process as demonstrated by mouse models, where ADAR1 knockout is embryonically lethal [1,2] and knockout of ADAR2 results in epilepsy and premature deaths [3]. In molecular aspects, A-to-I editing increases both transcript and proteome diversities, as inosine is decoded as guanosine by general cellular machineries (Figure 1). A-to-I editing in exonic regions of mRNAs can alter the coding sequence and consequently introduce amino acid substitutions in the protein [4,5]. In addition, editing affects splicing by creating or abolishing pre-mRNA splice sites [6,7] and suppresses RNAi by either repressing miRNA processing [8] or editing miRNA precursors [9,10]. Besides introducing nucleotide substitution, inosine itself confers distinct functional properties through interaction with inosine-specific binding proteins [11,12]. Hyperedited transcripts are found to be retained in the nucleus through binding to nuclear inosine-specific binding proteins, such as p54^nrb^ [11]. In addition, human endonuclease V, a ribonuclease specific for inosine-containing RNA, promotes degradation of edited transcripts [13].

**Figure 2:**
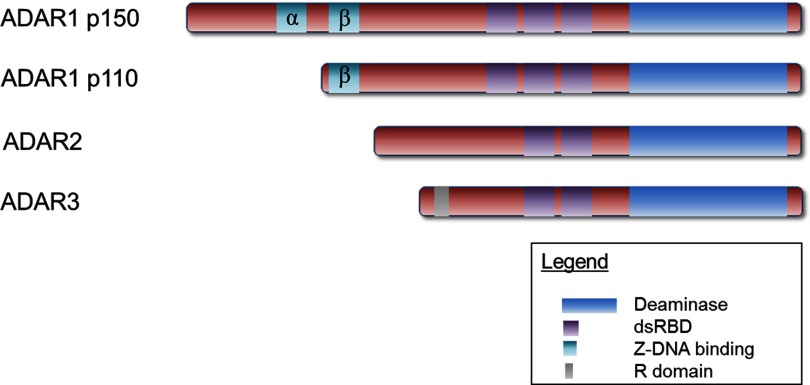
Domain architecture of mammalian ADAR family members There are three ADARs, ADAR1, ADAR2 and ADAR3, present in vertebrates. The ADARs share similar domain organization, including a conserved C-terminal deaminase domain (in blue) and variable numbers of dsRBDs (in purple). There are two predominant ADAR1 isoforms, p150 and p110, which are either constitutively expressed or inducibly expressed by interferon respectively. In addition, zDNA-binding domains (in green) are an exclusive feature of ADAR1 isoforms, whereas the R domain (in grey), an arginine/lysine-rich motif that binds ssRNAs, is unique to ADAR3.

Mammalian ADAR family comprises three highly conserved members namely ADAR1, ADAR2 and ADAR3. ADAR members possess a conserved C-terminal deaminase domain and variable numbers of dsRNA-binding domains (dsRBDs) [14] (Figure 2). Exclusively, the two ADAR1 isoforms have either one or two zDNA-binding domains that recognize left-handed helical DNA, an atypical structure associated with active transcription [15–18]. In contrast, ADAR3 contains a unique arginine/lysine-rich motif (R domain) that allows it to bind ssRNA substrates [19].

The current consensus is that only ADAR1 and ADAR2 are catalytically active [20,21] and they exhibit a certain degree of overlapping substrate specificity [22]. In contrast, ADAR3, a less studied ADAR member, has been described to be catalytically inactive since purified ADAR3 failed to edit known ADAR substrates such as glutamate-gated ion channel receptor (GluR-B) and serotonin-2C receptor (5HT_2C_R) transcripts *in vitro* [19]. Remarkably, there remains to be no *in vivo* evidence to prove beyond reasonable doubt that ADAR3 does not have any capacity to edit hitherto unknown ADAR substrates. With the advancements in RNA-sequencing technology, *in vivo* studies aiming to investigate the editing role of ADAR3 on an unbiased and global scale may come to fruition in the near future.

Intriguingly, previous observations noted that ADAR expression levels do not always correlate well with editing frequencies [23,24]. Therefore, it is evident that transcriptional regulation of ADARs is not the sole mode of regulation and additional regulatory mechanisms may exist on post-transcriptional, translational and post-translational levels. However, to date, the precise regulatory mechanisms governing this critical process have only been partially elucidated and the current state of knowledge will be reviewed herein.

## THE CASE FOR ADARs: ISOFORMS, SUB-CELLULAR LOCALIZATION AND REGULATION

### ADAR1

ADAR1 exists in two predominant isoforms, p110 and p150, generated through transcription from alternative promoters [25]. As depicted in Figure 2, these two isoforms share general structural similarities except that the N-terminally-extended p150 isoform comprises an additional zα-DNA-binding domain, resulted from usage of the translational initiation signal on exon 1 [25]. In contrast, the shorter p110 isoform utilizes the downstream AUG296 codon on exon 2 for translational initiation [26]. Unlike p110 isoform, which expression is driven by a constitutively active promoter, p150 expression is regulated by an interferon-inducible promoter [26]. Consequently, p150 expression is closely affected by host innate immune state as activated immune defence mechanism augments p150 expression through elevated interferon level [25,26]. In congruence, a recent study involving intracranial injection of a neurotropic strain of reovirus infection was found to induce ADAR1 p150 expression; however, it should be noted that this change in ADAR1 p150 level did not result in corresponding changes in site-specific A-to-I editing events [27].

In the cell, trans-acting regulators determine the sub-cellular localization of ADAR1 and the p110 isoform localizes predominantly in the nucleus [25,28]. Transportin-1, an import receptor, mediates nuclear localization of ADAR1 by recognizing the nuclear localization signal (NLS) within the third dsRBD of ADAR1 [29,30]. Interestingly, although ADAR1 p110 is devoid of the nuclear export signal (NES), exportin-5 is still able to export it from the nucleus to the cytoplasm [29,31]. In contrast, p150 isoform exists mainly in the cytoplasm as it contains a chromosomal region maintenance 1 (Crm1)-dependent NES on its exclusive N-terminal zα-domain [32,33]. Within the nucleus, p110 isoform shuttles between the nucleus and nucleolus and such a sub-nucleolar localization is highly dynamic and dependent on the presence of editable substrates to recruit ADAR1 p110 back into the nucleoplasm to catalyse editing [34]. Given that p110 isoform is found predominantly in the nucleus, it is mainly restricted to editing substrates present within the nucleus. Conversely, the ability of p150 isoform to shuttle between nucleo-cytoplasmic compartments allows it to catalyse editing of substrates present in both the cytoplasm and the nucleus [35]. ADAR1 p150 expands the pool of editable substrates, to include even cytoplasmic transcripts like viral RNAs.

ADAR1 expression is regulated on a post-transcriptional level by miRNAs, a class of small non-coding RNAs that regulate gene expression on the post-transcriptional level. In general, miRNAs, together with other players in the RNAi pathway, negatively regulate gene expression either by repressing translation or by promoting transcript degradation. miRNAs are approximately 22 nts in length and are at least partially complementary to the target sequences. In metastatic melanoma, it has been found that ADAR1 expression is frequently down-regulated by miR-17 and miR-432 [36].

Intriguingly, ADAR1 exerts both pro-viral and anti-viral functions. Through the course of evolution, some viruses have evolved and acquired advantageous mechanisms favouring their own replication. For instance, adenovirus encodes two non-coding, virus-associated (VA) RNAs, known as VAI and VAII. The major form, VAI RNA, antagonizes deaminase activity of ADARs and may alter viral and cellular gene expression, mainly through modulation of RNA editing [37].

ADAR1 has also been shown to be post-translationally modified through SUMOylation. This is a highly dynamic and reversible post-translational modification that involves covalent addition of small ubiquitin-like modifier (SUMO) on to the target protein. SUMOylation is involved in a myriad of cellular processes, including apoptosis, cell cycle regulation, protein stability and transcriptional regulation [38]. Human ADAR1, but not ADAR2, can be SUMOylated on lysine residue 418 [39]. SUMOylation on Lys^418^ attenuates ADAR1 editing activity without affecting its proper sub-cellular localization. It is hypothesized that SUMOylation of Lys^418^, a residue between the zDNA-binding domain and the first dsRBD of ADAR1, attenuates its editing activity through stereochemically hindering the binding of dsRNA and homodimerization of ADAR1, which are prerequisites for editing activities.

### ADAR2

ADAR2 gene expression can be stimulated by cAMP responsive element-binding protein 1 (CREB1) [40]. In addition, various alternatively-spliced ADAR2 isoforms have been reported [41–44]; however, only two distinct isoforms, ADAR2a and ADAR2b, possess disparate catalytic activities. ADAR2a and ADAR2b are generated through alternative splicing at the 3'-end of the pre-mRNA sequence encoding the deaminase catalytic domains [41]. The inclusion of additional 120 nts between the second and third putative zinc-co-ordination motifs in ADAR2b isoform [41,42] renders it less active as compared with ADAR2a [41].

ADAR2 negatively auto-regulates its activity through self-editing of its transcripts [6]. ADAR2 catalyses an A-to-I editing on its own pre-mRNA, converting an intronic AA dinucleotide into an AI dinucleotide which mimics the signature AG sequence commonly found on 3′ splice junctions. This self-editing event introduces an alternative proximal 3′-splicing acceptor site, adding 47 nts to the ADAR2 coding region. Insertion of this 47-nt cassette introduces a frameshift that gives rise to truncated ADAR2 proteins, unless a downstream, in-frame translational initiation codon is being used. However, the translational initiation efficiency of this internal initiation codon is low and hence little functional ADAR2 proteins can be translated from self-edited ADAR2 transcripts. In agreement, transgenic mice lacking ADAR2 self-editing capabilities have significantly higher ADAR2 protein levels concomitant with elevated editing frequencies of various ADAR2 substrates [42].

ADAR2 sub-cellular localization is determined by trans-acting regulators. ADAR2 has a prominent nuclear localization characteristic as it lacks NES and contains a non-canonical NLS within the first 64 amino acid residues [34]. Phosphorylation-dependent prolyl-isomerase (Pin1) functions as a positive regulator of ADAR2 by ensuring proper nuclear localization of ADAR2. In Pin1^−/−^ mouse, ADAR2 was mislocalized in the cytoplasm of embryonic fibroblasts, which resulted in reduced editing at the GluR2 glutamine/arginine and arginine/glycine sites [45]. This evidence shows that nuclear localization of ADAR2 is essential for adequate editing frequencies. Besides Pin1, nuclear import of ADAR2 is further modulated by importin α4 and α5 [46].

Similar to ADAR1, ADAR2 shuttles dynamically between the nucleus and nucleolus and the presence of editable substrates initiates its shuttling between the two compartments [34]. In the absence of editable substrates, ADAR2 is sequestered into the nucleolus by binding to nucleolar-enriched rRNA [47]. Intriguingly, the association between ADAR2 and rRNA is only transient and non-functional as these rRNA are not edited despite having significant binding to ADAR2. This could represent a regulatory mechanism that prevents aberrant editing activities by maintaining low concentrations of ADAR2 at its active site when there are limited editable substrates and yet maintaining a readily available pool of functional ADAR2 to cope with a sudden influx of substrates that need to be edited.

Excitotoxic level of glutamate induces specific ADAR2 proteolytic cleavage between the two dsRBDs [48]. This proteolytic cleavage requires activation of *N*-methyl-D-aspartate (NMDA) receptor which then initiates a cascade of events to ultimately activate the effector, calpain protease. The cleaved ADAR2 is now rendered non-functional due to its inability to bind to dsRNA substrates. The physiological impact of ADAR2 cleavage is pronounced. The decrease in GluRA2 glutamine/arginine editing causes a high calcium influx and excitotoxic neuronal death. However, it remains to be determined whether proteolytic cleavage of ADAR2 is a common mode of regulation in other non-neuronal cell types.

Other factors that regulate ADAR2 have also been described. The crystal structure of ADAR2 unveiled a unique feature of its catalytic domain. A molecule of inositol hexakisphosphate (IP6) was found to be buried within the enzyme core of ADAR2 and is essential for proper protein folding and enzymatic activity of ADAR2 [49].

Besides affecting nuclear localization of ADAR2, Pin1 also enhances the stability of ADAR2 proteins. ADAR2 mutants that can no longer interact with Pin1 were found to be less stable and the presence of Pin1 delays ADAR2 degradation [45]. In contrast to Pin1, the E3 ubiquitin ligase WW domain-containing protein 2 (WWP2) destabilizes ADAR2 by catalysing its ubiquitination and subsequent degradation [45].

A heterologous editing assay that screens for editing regulators in yeast allowed the identification of ribosomal protein S14 (RPS14), Serine/Arginine-rich splicing factor 9 (SFRS9) and DEAH box polypeptide 15 (DDX15). Although the detailed regulatory mechanisms adopted by these proteins have not been studied, it has been proposed that the landscape of ribonucleoproteins (RNPs) affects RNA editing [50].

## CROSS-TALKING OF ADAR1 AND ADAR2 WITH ADAR3, THE THIRD ADAR MEMBER

Homodimerization of ADARs is necessary for RNA editing activity [51,52]. However, the possibility of heterodimerization of ADAR members has been a matter of controversy, with confounding experimental observations made by different research groups [19,53]. FRET analysis in HeLa cells subsequently showed that ADAR1 and ADAR2 do interact, albeit with a low efficiency [53]. Further, studies showed that each ADAR member can attenuate or modulate the editing activity of other members [19,54,55]. Presumably, the antagonistic relationship between ADAR1 and ADAR2 is due to the formation of non-functional heterodimers [53] and/or the non-productive competition for substrates [54].

ADAR3 remains to be a peculiar member of ADARs as it has no demonstrable editing activity *in vitro* [19]. As mentioned earlier, the precise roles of ADAR3 in RNA editing have not been fully elucidated. Plausibly, ADAR3 may utilize its exclusive N-terminal arginine-rich motif, which is thought to recognize and bind to specific stem-loop structures of ssRNA substrates, to target a selected set of substrates. Notably, *in vitro* experiments demonstrated that ADAR3 effectively suppresses editing of 5HT_2C_R RNA by ADAR1 and ADAR2 [19]. However, the mechanism of this suppression remains unknown. This observation supports the hypothesis that ADAR3 might play a negative regulatory role in A-to-I editing. Heterodimerization of ADAR3 with either ADAR1 or ADAR2 might render ADAR1 and 2 inactive.

Capitalizing on the probable regulatory role of ADAR3 in A-to-I editing, it may be useful to understand the sub-cellular localization of this protein. Sub-cellular localization of ADAR3 is mostly nuclear as importin α1 specifically recognizes the R-motif to mediate nuclear transport [46]. Till now, due to the paucity in our understanding of ADAR3, it is unclear whether importin α1-mediated nuclear import has any contributing roles to ADAR3 functions.

## SPECIES-, TISSUE- AND CELL-SPECIFIC A-TO-I EDITING REGULATIONS

RNA editing is a process under dynamic regulation. High throughput transcriptome sequencing revealed partially distinct A-to-I editing landscapes in brains of humans, chimpanzees and macaques [56]. Despite the distinct editing landscapes, certain editing sites are evolutionarily conserved. It has been proposed that conservation of specific editing sites between related species requires simultaneous conservation of cis-elements, especially the elements providing the essential RNA structures for ADAR recognition [57,58]. However, no species-specific transregulator has been identified to be responsible for the distinct A-to-I editing landscapes in related species.

Spatiotemporal regulation of A-to-I editing further complicates the regulatory network in cells [59–61]. Specific tissues modulate their ADAR2 protein levels through tissue-specific alternative splicing of the ADAR2 pre-mRNA. Inclusion of exon 7a generates in-frame premature termination codons which are inducers of non-sense-mediated mRNA decay (NMD), a surveillance pathway to degrade mRNAs with premature termination codons. This selective splicing event allows tissues with low basal editing levels to down-regulate their ADAR2 levels on a post-transcriptional level [62]. Capitalizing on the fact that certain sites are specifically edited in particular tissues, or in a precise spatiotemporal manner, strongly suggests the existence of regulators that are currently unknown.

## PERTURBED A-TO-I EDITING REGULATION AND DISEASES

Given the prominent effects of A-to-I editing on transcriptome, it is not surprising that aberrant A-to-I editing contributes to various diseases. Pioneering studies mainly focused on neurological functions, development and diseases, as A-to-I editing was first found to be a widespread process in the mammalian brain. These individual studies revealed that transcripts of numerous neurotransmitter receptors are precisely edited and dysregulated editing results in neurological diseases such as amyotrophic lateral sclerosis (ALS), transient forebrain ischaemia, Prader–Willi syndrome and psychiatric disorders [63]. Besides, perturbed ADAR activities also contribute to other diseases.

Recently, ADARs have emerged as promising therapeutic targets in cancer, a disease that is thought to arise from accumulation of various driver mutations that favour cell survival and proliferation. In the past, attention was on genomic mutations. However, increasing evidence demonstrates inconvertibly that mutations on the transcriptome level, owing to dysregulated RNA editing activities, have similar pathological effects. To date, perturbed A-to-I editing has been shown to be involved in various cancers, including acute leukaemia, breast cancer, hepatocellular carcinoma and neuroblastoma [63].

The tight link between dysregulated A-to-I editing and diseases highlights the essentiality for a balanced regulation and regulators responsible for maintaining the balance are promising therapeutic targets.

## CONCLUSION AND PERSPECTIVE

**Figure 3:**
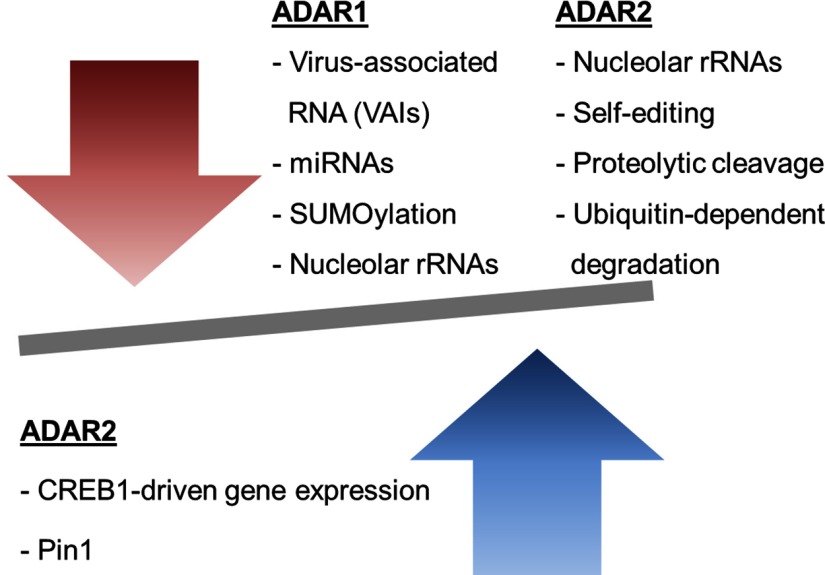
Known regulatory mechanisms in A-to-I editing Regulation of A-to-I editing is multifaceted. ADAR1 has been shown to be negatively regulated by VA RNA (VAIs), miRNAs, SUMOylation and nucleolar rRNAs. ADAR2 is down-regulated by nucleolar rRNAs, self-editing, proteolytic cleavage and ubiquitin-dependent degradation. Conversely, ADAR2 gene expression can be up-regulated by CREB1 and Pin1 ensures proper localization of ADAR2 in the nucleus and delays ADAR2 degradation.

The precise underlying regulatory mechanism of A-to-I editing has only been partially elucidated. In the present review, we reinforced the notion that ADARs, like many other crucial cellular components, are tightly regulated on transcriptional, post-transcriptional and post-translational levels (Figure 3). Although ADAR1 and ADAR2 are the sole factors needed to catalyse RNA editing *in vitro* [64,65], it is apparent that the activity and selectivity of ADARs in cells are precisely regulated in a multifactorial manner. They are, perhaps, governed by an editosome complex, comprising numerous essential regulators. The factors determining substrate selectivity of ADARs have been a challenging conundrum for researchers in the field of A-to-I RNA editing. The lack of RNA sequence similarity in ADAR substrates strongly suggests that substrate structure plays a more pivotal role in determining substrate specificity of ADARs. Building on this, it is highly plausible that RNA helicases may have regulatory functions in A-to-I editing, through remodelling of the substrate structures.

Thus far, little advances have been made to investigate the substrate structures as RNA structome, the study of RNA structures, is technically challenging. With the introduction of selective 2′-hydrox acylation analysed by primer extension (SHAPE), coupled with RNA-sequencing technique, it might now be feasible to probe for *in vivo* RNA structures in a high throughput manner [66–68].

In sum, our knowledge of A-to-I RNA editing is steadily increasing. Comprehensive delineation of the regulatory mechanisms underlying this pivotal process might eventually prove to be beneficial, especially in translational research. Promisingly, the invention of methods to correct specific RNA editing events [69–71] has opened up possibilities of devising novel therapeutic strategies to ameliorate diseases caused by aberrant A-to-I editing.
